# Assessing the utility of the COVID-19 epidemic Situations of Concern classification system in guiding operational responses to the pandemic in the WHO African region: retrospective analysis

**DOI:** 10.3389/fpubh.2025.1562525

**Published:** 2025-07-24

**Authors:** Opeayo Ogundiran, Jessica L. Abbate, Sooyoung Kim, Mamadou Saliou Kalifa Diallo, Michel Muteba, Daniel Cardoso Portela Camara, Lucas Bianchi, Thierno Balde, Boniface Oyugi, Ann Fortin, Jayne Baykika-Tusiime, George Sie Williams, Franck Mboussou, Charles Okot, Freddy Mutoka Banza, Kabego Laundry, Ephraim Nonso Ejiofor, Trevor M. Kanyowa, Rashidatu Kamara, Phionah Atuhebwe, Nicksy Gumede, Belinda Loiuse Herring, Solomon Woldetsadik, Joseph Okeibunor, Etien Koua, Dick Chamla, Fiona Braka, Abdou Salam Gueye

**Affiliations:** ^1^World Health Organization Regional Office for Africa, Brazzaville, Republic of Congo; ^2^Geomatys, Montpellier, France; ^3^New York University School of Global Public Health, New York, NY, United States; ^4^Scientific Computing Program - Oswaldo Cruz Foundation, FIOCRUZ, Rio de Janeiro, Brazil; ^5^Centre for Health Services Studies (CHSS), University of Kent, Canterbury, United Kingdom; ^6^World Health Organization Country Office, Abuja, Nigeria; ^7^World Health Organization Country Office, Asmara, Eritrea; ^8^World Health Organization Country Office, Harare, Zimbabwe; ^9^UNICEF Headquarters, Nairobi, Kenya

**Keywords:** COVID-19, epidemic, SAR-CoV-2, Situation of Concern, WHO African region

## Abstract

During a public health emergency, early implementation of response activities is crucial for saving lives and protecting livelihoods. The COVID-19 pandemic, declared by the World Health Organization (WHO) on March 11, 2020, posed a global public health crisis that required timely decision-making despite limited data and capacity. In this context, WHO’s Regional Office for Africa (AFRO) developed the Situations of Concern (SOC) classification system to assess and monitor epidemiological risk across its 47 Member States. We conducted a retrospective analysis to evaluate the performance and operational utility of the SOC system. Using weekly country-level COVID-19 surveillance data, we found that the system demonstrated strong alignment with epidemic wave patterns, with a sensitivity of 83% and specificity of 88%. SOC classifications supported timely operational decision-making in over 70% of documented support instances. Effective management of limited resources through SOC assessments also helped ensure fair distribution of support across communities. Our findings suggest that adaptable classification systems like SOC can provide effective decision-support under conditions of limited data availability, improving outbreak preparedness and response in resource-constrained settings.

## Introduction

The COVID-19 pandemic, caused by the sudden emergence of SARS-CoV-2 from an animal reservoir (1), posed unprecedented challenges for modern health emergency response systems worldwide (2). At the onset of the pandemic, with response systems still being established and the potential impact of the virus largely unknown, countries implemented sweeping measures such as travel bans and the interruption of major societal functions. These actions, while necessary to curb transmission, led to significant economic and social disruptions (3–5). In addition to these technical interventions, many countries emphasized behavioral non-pharmaceutical interventions (NPIs)—such as mask-wearing, hand hygiene, and physical distancing—sometimes referred to as “behavioral vaccines” (6). These practices played a vital role in reducing transmission regardless of geographic or socioeconomic context. In the World Health Organization (WHO) African Region (AFR), where many Member States (MS) had limited preparedness capacities, reliance on such blanket measures was pronounced. In contrast, countries with more advanced capacities were able to lift restrictions earlier, leveraging strategies such as widespread testing to limit viral spread (7–9).

Decisions regarding when and how to act, as well as how to allocate limited resources, were critical in containing SARS-CoV-2 transmission. By 2021, the second year of the pandemic, most AFR Member States had emerged from initial lockdowns. However, new waves of COVID-19 driven by variants with distinct transmissibility and severity profiles began affecting populations at different times (10). One of the core responsibilities of the WHO Regional Offices during the pandemic was to rapidly assess the needs of Member States, make evidence-based recommendations for response activities, and support these efforts through resource mobilization. Although individual countries maintained their own monitoring and evaluation systems for national and sub-national operations, these systems varied widely in their objectives, data quality, and ability to account for unique local contexts. Crucially, no unified system existed to enable cross-country assessment of the pandemic’s impact within a region while accounting for such variations. This gap highlighted the need for a regional operational classification system capable of informing decisions about scaling response activities up or down in line with each country’s changing epidemiological landscape. While a global system was under development by a WHO Headquarters team in Geneva to coordinate efforts across all six regions (11), the Regional Office for Africa (AFRO) developed an interim system tailored to the immediate needs and specific contexts of its 47 Member States.

In June 2021, the Incident Management Support Team (IMST) for the AFR Regional COVID-19 response introduced a set of indicators using basic epidemiological data from Member States to track the pandemic’s trajectory. These indicators, collectively termed Situations of Concern (SOC), enabled systematic classification of Member States into epidemic categories (“low incidence,” “very high incidence,” “alert,” and “resurgence”) based on national disease burden and public health impact. Classification assignments were informed by criteria such as recent trends in new cases and deaths, incidence per capita, COVID-19 test positivity rates, and vaccination coverage. These indicators were assessed weekly to categorize each Member State, providing a foundation for operational decision-making regarding resource allocation.

Following the emergence of the SARS-CoV-2 Omicron variant and subsequent changes in transmission dynamics, the SOC indicator criteria were adjusted in September 2022 (12). These adjustments allowed the system to remain relevant and operationally useful, and several Member States adopted the SOC classification for subnational early warning and decision-making. At the global level, the classifications informed international efforts to monitor and prioritize epidemic situations across WHO regions (11).

Retrospective evaluations of decision-support systems are essential to assess their real-world impact, identify opportunities for improvement, and inform the design of future outbreak preparedness tools (13). Although future pandemics may differ from COVID-19, many of the systemic, logistical, and information-related challenges observed are likely to recur, particularly in resource-constrained settings (14).

This study retrospectively reviews the implementation of the WHO AFR’s SOC indicator-based decision-making system. It evaluates the system’s utility as an early warning and decision-making tool and its effectiveness in raising timely alerts about concerning epidemic situations across WHO AFR Member States. Conducting this retrospective analysis provides a critical opportunity to learn from past experience. Understanding the strengths and limitations of the SOC classification during the COVID-19 pandemic can inform the design of future surveillance systems and decision-making tools—especially in regions with limited resources and high epidemiological uncertainty.

## Methods

### Study design

This study utilized a retrospective analysis to assess the operational utility of the Situation of Concern (SOC) classification system in predicting and signaling observed COVID-19 trends across WHO African Region Member States. The analysis period covered the period from Epidemiological Week (EW) 23 of 2021 (June 7, 2021) to EW 6 of 2023 (February 6, 2023), corresponding to the timeframe during which the SOC classifications were implemented and used to guide operational decisions.

### Participants

#### Inclusion criteria

All 47 Member States within the WHO African Region (15) were eligible for inclusion in the analysis. These Member States submitted COVID-19 surveillance data to the WHO Regional Office for Africa in compliance with the International Health Regulations (2005) (16). Each Member State served as an individual unit for SOC classification and epidemic wave assessment.

### Procedure

#### Definitions

Confirmed COVID-19 cases were defined as individuals testing positive for SARS-CoV-2 by polymerase chain reaction (PCR) or antigen rapid diagnostic test (AgRDT), meeting either probable or suspected case definitions per WHO guidelines, or as an asymptomatic contact of a probable or confirmed case with a positive test result (17). COVID-19 deaths were defined as fatalities attributable to a clinically compatible illness in probable or confirmed COVID-19 cases, excluding deaths caused by unrelated factors (17).

National epidemic waves were identified retrospectively by examining weekly incidence trends. A COVID-19 epidemic “wave” was defined as a period of sustained and significant week-on-week increases in new cases, followed by a decline until weekly incidence returned to pre-wave levels. Wave events were characterized by two milestones. The “start” week was the epidemiological week when cases began rising, and the “peak” week was the week with the highest incidence, signifying the highest transmission period of SARS-CoV-2. For irregular data reporting, a 3-week moving average of incidence rates was used to determine peaks.

#### Data source

Primary epidemiological data on confirmed COVID-19 cases, deaths, testing rates, and vaccination coverage were obtained from Member State submissions to WHO’s Regional Office for Africa and published on the WHO’s global COVID-19 dashboard (18), in compliance with International Health Regulations (2005) (16). The data were aggregated by epidemiological week (Monday through Sunday) according to international standards of epidemiological data reporting (19).

#### COVID-19 Situation of Concern (SOC) classification

SOC classifications were assigned weekly to Member States based on the defined criteria. Initially, three criteria were used:

(i) A 20% or more increase in the weekly incidence or mortality over two consecutive weeks (or over four consecutive weeks for irregular reporting).
(ii) A 30% or more increase in weekly incidence compared to the previous peak and.
(iii) A weekly incidence ratio of 500 or more cases per 100,000 population.

A Member State was classified as being in “Resurgence” for the week under review if it met the first two criteria. For those classified as “Alert,” at least one of the first two criteria had to be met. Member States meeting only the third criterion were categorized as having “Very High Incidence.” If none of the criteria were met, the Member State was classified as “Low Incidence,” even if no new cases were reported. In cases where sufficient data were unavailable by the evaluation date, the existing classification was retained.

In September 2022, WHO AFRO began revising the methodology for the SOC classification in response to the unprecedented surge in COVID-19 cases and the altered transmission dynamics associated with the emergence of the Omicron variant. This period was marked by very high transmission rates and incidence levels, coupled with relatively lower mortality compared to earlier phases of the pandemic. During this transitional phase, Alert classifications were issued for suspected new epidemic events based on contextual assessments and patterns observed during pre-Omicron waves, even when official criteria were not met. However, Resurgence classifications were used less frequently due to uncertainty and challenges in maintaining consistent messaging while the criteria were under review.

By Epidemiological Week 1 of 2023, the following revised criteria were officially adopted for SOC classifications:

(i) A 20% or more increase in transmission indicators such as per-capita incidence ratio or test positivity rate (TPR).
(ii) A 20% or more increase in severity indicators such as new hospital admissions or mortality.

**Figure 1:**
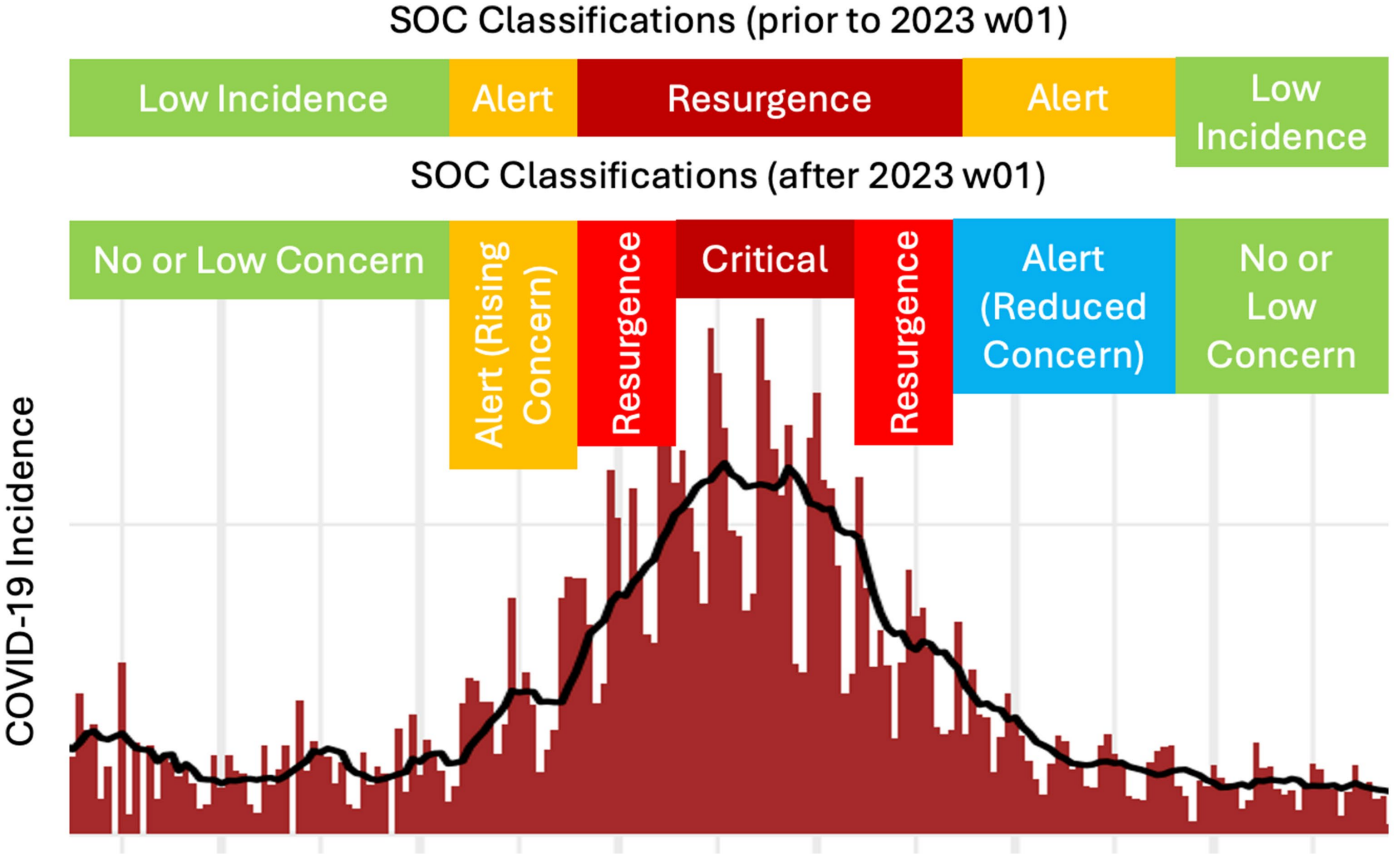
COVID-19 epidemic Situation of Concern (SOC) alert classifications as they relate to observed case incidence (here, shown for daily timesteps).

An “Alert (Rising) Concern” classification was issued when one of the two parameters increased over a two-week period. “Resurgence Concern” was assigned if one parameter increased over a three-week period. “Critical Concern” classification was designated if both parameters increased over a three-week period. An additional phase was introduced to the classification to account for an “Alert (Reduced) Concern,” to classify Member States showing a decline in either parameter over a two-week period. If none of the parameters were met, the Member State was classified as being of “No or Low concern” (Figure 1).

### Data analysis

#### Calculation of epidemic indicators

The percentage change in incidence and mortality were computed as the week-on-week percentage difference in confirmed COVID-19 cases and deaths, respectively, (20). The test positivity rate was calculated as the proportion of positive tests among all SARS-CoV-2 tests conducted (21). The per-capita incidence ratio was expressed as the number of new cases per million population of the Member State (22). The vaccination coverage was calculated as the proportion of individuals who received at least one dose of a COVID-19 vaccine, using UN population estimates as the denominator (23, 24). Data analysis was conducted using R (version 4.0.3) (25).

#### Evaluation of the operational utility of the SOC classification

The operational utility of the SOC classification system was evaluated based on five hypotheses focusing on the ability of Alert and Resurgence classifications to signal or predict the growing or re-emerging burden of COVID-19. The analyses assessed:

(1) The capacity of *Alert* or *Resurgence* classifications to signal the occurrence of an epidemic wave. This hypothesis addresses the specific purpose for which the SOC classification system was designed. The following hypotheses test for additional utility of the system by addressing timeliness and adaptability of the classifications.
(2) The ability of *Alert* classification to signal the start of a wave.
(3) The ability of *Alert* classification to predict or signal the peak of a wave.
(4) The ability of *Resurgence* classification to predict or signal the peak of a wave.
(5) The impact of Omicron-driven changes on classification utility, tested by evaluating performance pre- and post-criteria adjustments.

The term *predict* refers to instances where an SOC classification (Alert or Resurgence) was issued at least 2 weeks prior to the event of interest, providing early notice. In contrast, *signal* refers to SOC classifications issued within 2–3 weeks of the event, indicating an imminent or ongoing situation. Both measures were designed to evaluate how effectively the SOC indicators could guide preparedness and resource allocation within the WHO African region.

The evaluation of operational capacity began with an analysis of the ability of SOC classifications to signal the occurrence of a COVID-19 wave. This involved examining the success of issuing an *Alert or Resurgence* classification during periods extending from 3 weeks before the start of a wave to 3 weeks after its end. Recorded weekly SOC classifications were cross tabulated with documented epidemic wave events to compute performance metrics, including sensitivity, specificity, and positive predictive value (PPV).

The operational utility of the SOC classifications in predicting and signaling the start and peak of COVID-19 waves in a timely manner were then evaluated. Predictive ability was evaluated by determining whether an *Alert or Resurgence* classification was issued at least 2 weeks prior to the wave’s peak. For signaling ability, performance metrics (sensitivity, specificity, PPV) were calculated for Alert classifications issued within 3 weeks before or after the start of the wave; and for *Alert or Resurgence* classification issued within 2 weeks before or after the peak of the wave. Instances where no *Alert* or *Resurgence* classification was issued during the specified epidemic event periods represented false negatives (FNs), while false positives (FPs) referred to cases where an *Alert* or *Resurgence* classification was issued outside of these periods. FN and FP classifications were based on the hypotheses under evaluation and did not necessarily indicate errors in classification. In instances where epidemic events occurred in close succession, a single classification could align with the detection window of one wave while falling outside the window for another. To prevent counting a single alert as both a true and false positive, we conservatively classified such cases as true positives. This approach acknowledges the alert’s relevance in signaling ongoing or imminent transmission.

#### Time-stratified analysis

**Figure 2:**
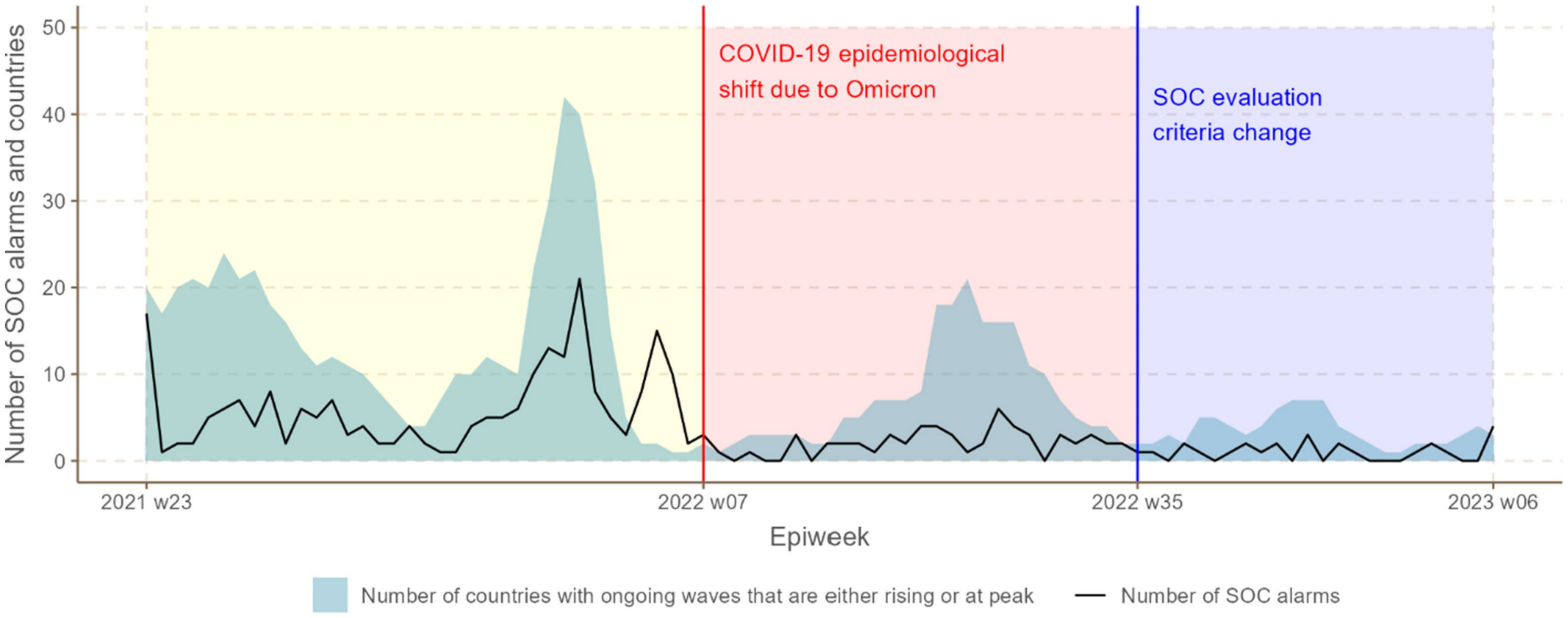
Temporal trends in number of SOC epidemic concern classifications (*Alert or Resurgence* classification) and epidemic waves over time stratified by time period during the COVID-19 pandemic.

To assess the impact of the September 2022 adjustments on the operational utility of the SOC indicators in the context of the Omicron variant’s emergence, the study period was segmented into three distinct time strata:

- T1: Epidemiological Week (EW) 23 of 2021 to EW 7 of 2022, representing the period prior to the Omicron-driven changes in COVID-19 dynamics.
- T2: EW 8 of 2022 to EW 35 of 2022, corresponding to the Omicron surge period during which no adjustments were made to the SOC system.
- T3: EW 36 of 2022 to EW 6 of 2023, encompassing the period after the modified SOC criteria were introduced in response to the Omicron variant (Figure 2).

The SOC classifications were reassessed for each period using the same analytical methods as the main analysis. Performance metrics, including sensitivity, specificity, and positive predictive value (PPV), were calculated and compared across the three time periods to assess differences in the system’s ability to predict and signal epidemic events. For this time-stratified analysis, the *Critical* Concern classification was categorized as a Resurgence classification, while only the *Alert (Rising Concern)* classification was considered as an *Alert* classification. The *Alert (Declining Concern)* classification was excluded from predictive and signaling performance analyses to maintain focus on rising epidemic trends.

## Results

### Descriptive analysis

**Figure 3:**
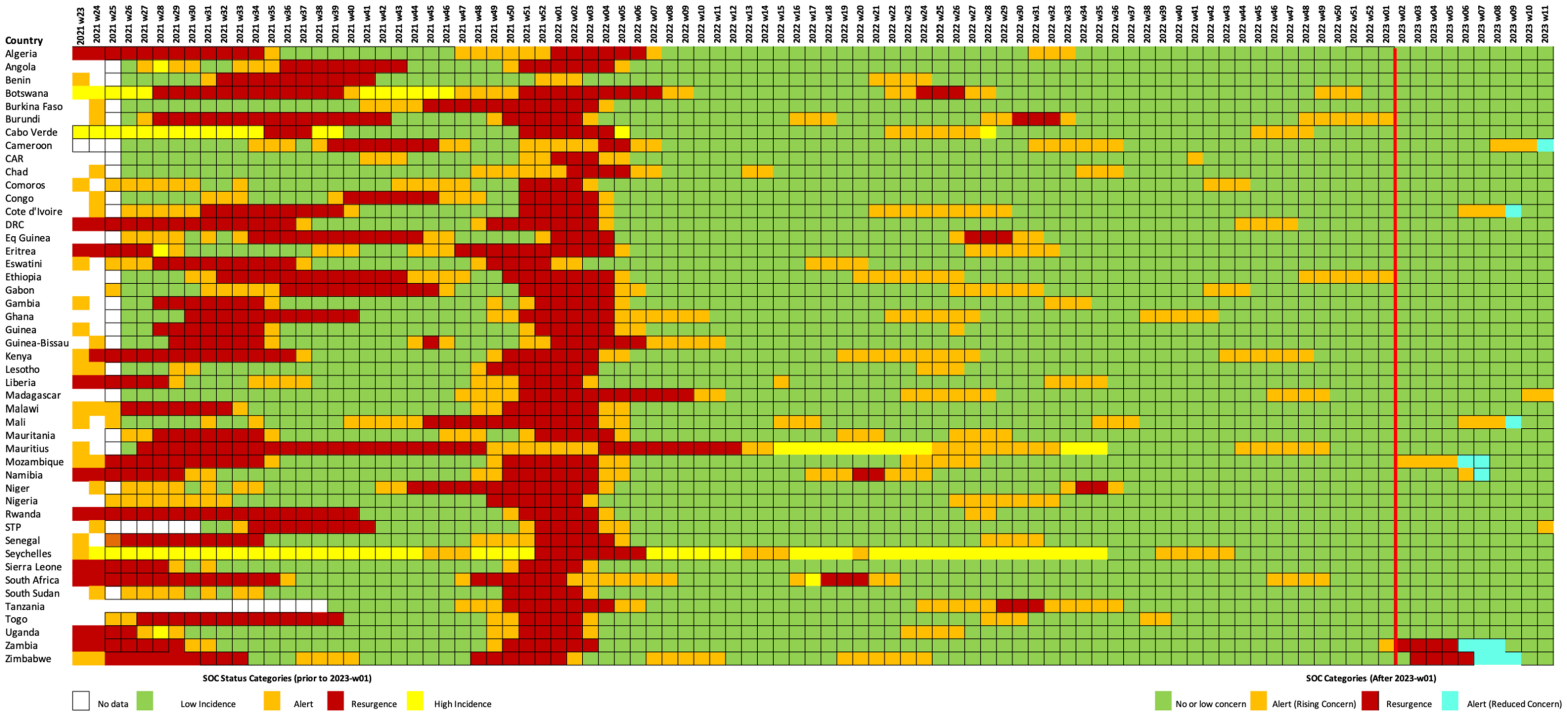
Weekly SOC classification of Member States from epidemiological week 23-2021 to week 11-2023. The red vertical line signifies when SOC criteria were revised to adapt to the shift in epidemiological characteristics provoked by emergence of the Omicron variant. The following colors signify the SOC classification in place for each week: Green = no or low concern, Yellow = Very High Incidence, Orange = Alert (rising or reduced concern, prior to revised SOC criteria; rising concern following revised SOC criteria), Red = Resurgence, and Blue = Alert (Reduced Concern), White = No classification.

**Figure 4:**
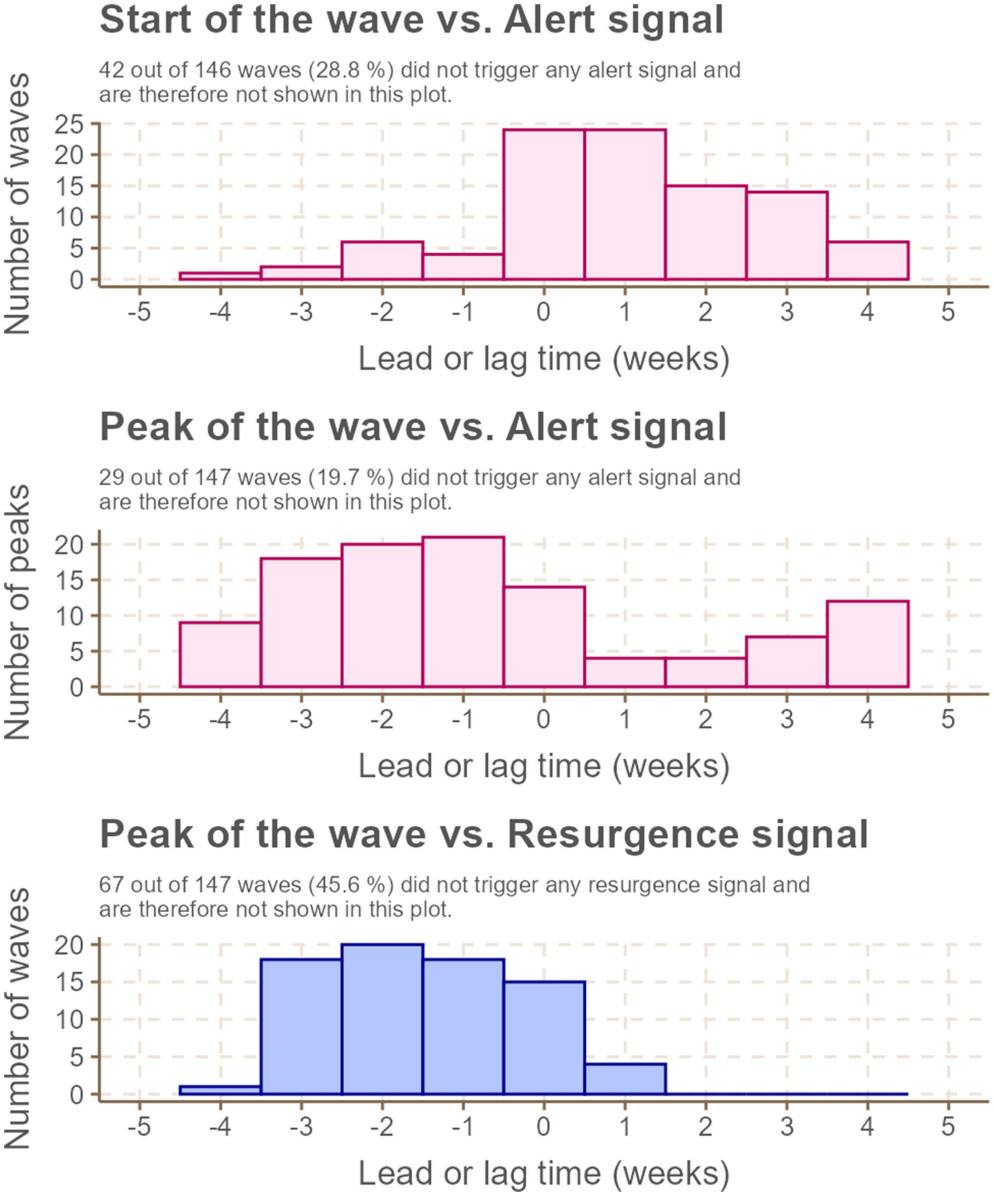
Lead (negative) and lag (positive) times (in weeks) for issuance of SOC Alert and Resurgence classifications compared to epidemic events (start or peak of national COVID-19 epidemic waves).

Over the 88 epidemiological weeks analyzed, the WHO African Region recorded 146 national COVID-19 epidemic waves across its 47 Member States, as depicted in Figure 2. During this period, WHO AFRO issued 98 *Resurgence* and 204 *Alert* classifications, illustrated in Figures 2, 3. The relationship between national COVID-19 waves and SOC epidemic concern classifications is detailed in Figure 4 further shows the distribution of SOC classifications in relation to the start and peak of COVID-19 epidemic waves.

Among the 146 COVID-19 waves observed during the study period, over half were associated with a *Resurgence* classification issued within 5 weeks before or after the wave’s start, and more than 70% were linked to an *Alert* classification issued within the same time frame. Of these waves, 26.0% had an *Alert* classification issued either prior to or during their start week, with a median lead or lag time of 0 weeks (interquartile range [IQR]: 0–2 weeks of lag). Regarding wave peaks, 56.5% had an *Alert* classification issued either preceding or concurrent with the peak, with a median lead time of 2 weeks (IQR: 1–3 weeks). Similarly, 51.0% of peaks were preceded or simultaneously accompanied by a *Resurgence* classification, also with a median lead time of 2 weeks (IQR: 1–3 weeks).

### Operational utility of the SOC classifications

Table 1 provides an overview of the operational utility of SOC *Resurgence* and *Alert* classifications in predicting and signaling national COVID-19 epidemic events. Overall, the SOC classifications demonstrated high specificity and low sensitivity for predicting epidemic events. Their performance was notably stronger in signaling epidemic events, as reflected by higher sensitivity and positive predictive value (PPV) metrics (Table 1).

**Table 1:** Summary of operational utility of the SOC classifications in comparison to the COVID-19 epidemic events (start and peak of national waves).

Utility measure		TP	TN	FP	FN	Sensitivity (95% CI)	Specificity (95% CI)	PPV (95% CI)
*Alert* classification vs. start of wave	Signaling (within +/− 3 weeks)	91	1768	71	44	67.4% (59.5–75.3%)	96.1% (95.3–97.0%)	56.2% (48.5–63.8%)
*Alert* classification vs. peak of wave	Signaling (within +/− 2 weeks)	64	1746	81	83	43.5% (35.5–51.6%)	95.6% (94.6–96.5%)	44.1% (36.1–52.2%)
	Predicting (at least 2 weeks prior to wave peak)	40	1,472	133	333	10.7% (7.6–13.9%)	91.7% (90.4–93.1%)	23.1% (16.8–29.4%)
*Resurgence* classification vs. peak of wave	Signaling (within +/− 2 weeks)	57	1819	24	90	38.8% (30.9–46.7%)	98.7% (98.2–99.2%)	70.4% (60.4–80.3%)
	Predicting (at least 2 weeks prior to wave peak)	39	1,552	59	344	10.2% (7.2–13.2%)	96.3% (95.4–97.3%)	39.8% (30.1–49.5%)

TP = True positive; TN = True negative; FP = False positive; FN = False negative; PPV = Positive predictive value; CI = Confidence interval.

More than 90% of the SOC classifications effectively signaled or predicted one of the two epidemic events (start or peak of a wave). Specifically, 67.4% of waves were signaled by epidemic concern classifications within 3 weeks of their start. However, the classifications exhibited comparatively lower effectiveness in signaling or predicting wave peaks (Table 1).

### Time-stratified analysis

**Figure 5:**
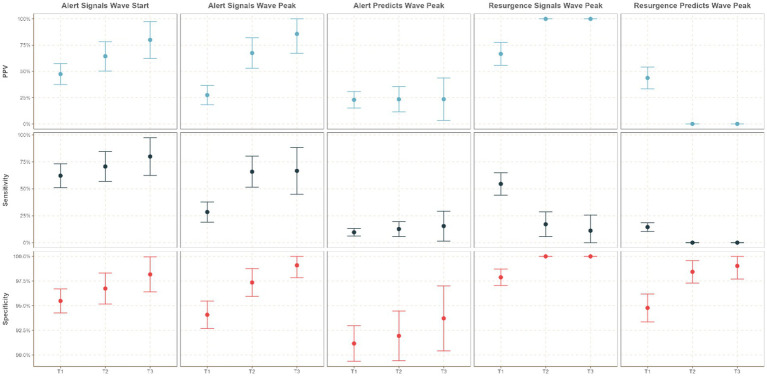
Time-stratified operational utility of the SOC epidemic concern classifications.

Figure 5 highlights changes in the operational utility of SOC indicators across three defined time strata.

The emergence of the Omicron variant during T2 significantly impacted the performance of the Resurgence classification, particularly in predicting and signaling wave peaks. During T2, the sensitivity of Resurgence classifications to predict wave peaks dropped to 0% (95% CI: 0–0%) with a PPV of 0% (95% CI: 0–0%), compared to T1, where sensitivity was 14.4% (95% CI: 10.3–18.6%) and PPV was 43.8% (95% CI: 33.5–54.1%). Similarly, the sensitivity of Resurgence classifications to signal wave peaks declined to 17.1% (95% CI: 5.6–28.6%) during T2, with a PPV of 100% (95% CI: 100–100%), compared to T1’s sensitivity of 54.5% (95% CI: 44.1–64.9%) and PPV of 66.7% (95% CI: 55.8–77.6%).

Despite the modifications to SOC classification criteria during T3, the sensitivity of the Resurgence classification did not improve. However, the two resurgence events that occurred during T3—in Zambia and Zimbabwe—were accurately captured using the revised SOC methodology.

In contrast, the operational utility of the Alert classification demonstrated resilience during the emergence of Omicron and showed notable improvement over time, particularly in signaling the start and peak of epidemic waves. During T2, the sensitivity of Alert classifications to signal the start of a wave increased to 70.7% (95% CI: 56.8–84.7%) with a PPV of 64.4% (95% CI: 50.5–78.4%), compared to T1’s sensitivity of 62.2% (95% CI: 51.1–73.2%) and PPV of 47% (95% CI: 37.5–57.4%). Similarly, the sensitivity of Alert classifications to signal wave peaks improved to 65.9% (95% CI: 51.3–80.4%) during T2, with a PPV of 67.5% (95% CI: 53.0–82.0%), compared to T1’s sensitivity of 28.4% (95% CI: 19.0–37.8%) and PPV of 27.5% (95% CI: 18.3–36.6%).

During T3, the Alert classification’s ability to signal epidemic events showed significant enhancement. The sensitivity to signal the start of waves within 3 weeks before or after increased to 80% (95% CI: 62.5–97.5%), with a PPV of 80% (95% CI: 62.5–97.5%). Additionally, the sensitivity to signal wave peaks within 3 weeks before or after rose to 67% (95% CI: 44.9–88.4%), with a PPV of 85.7% (95% CI: 67.4–100.0%). The sensitivity of Alert classifications to predict wave peaks within 3 weeks before or after also improved during T3, reaching 15.4% (95% CI: 1.5–29.3%), with a PPV of 23.5% (95% CI: 3.4–43.7%).

## Discussion

This study aimed to assess the implementation, operational utility, strengths, and limitations of the COVID-19 Situations of Concern (SOC) classification system in the WHO African Region. Our findings provide clear evidence on the system’s overall performance in signaling epidemic waves and guiding resource allocation, while also highlighting challenges related to data quality and contextual variability. The impact of evolving epidemiological dynamics, notably the Omicron variant surge, and subsequent system adaptations were critically evaluated. The following sections elaborate on these key themes, beginning with an overview of the SOC system’s performance.

### Overall performance of the SOC classification system

The SOC classification system was designed to inform decisions that support the adequate mobilization and distribution of resources to various Member States by the WHO Africa Regional Office during the acute phase of the COVID-19 pandemic. This approach aligns with evidence from the African Region showing that real-time situational analysis and early warning mechanisms were critical in shaping timely and effective COVID-19 responses (26). A retrospective review of the system’s predictive ability and operational utility revealed high specificity but low sensitivity in forecasting the start and peak of epidemic waves of COVID-19 in the African region. While both the Resurgence and Alert signals displayed specificity above 90%, the Alert signal exhibited the highest sensitivity in predicting the peak of the COVID-19 wave following the re-adjustment of SOC criteria and classifications after the emergence of the SARS CoV-2 Omicron variant.

### Challenges and contextual factors

The low sensitivity primarily stemmed from the inherent limitations of the SOC indicator criteria, which relied on the assessment of epidemiological developments in preceding weeks, often complicated by delayed and incomplete reporting. While the definition of an “epidemic wave” can vary across settings and remains a subject of debate in the scientific literature (27), our use of the term was operationally defined to support the structured application and evaluation of the SOC classification system within the WHO African Region. Consequently, as illustrated in Figure 3, most signals (52.6%) were issued with a median lag of 1 week (IQR: 0–3 weeks). In some instances, delays in reporting from Member States to the regional office exacerbated the problem of extended lags. For example, notable missed events in Chad and Lesotho underscored the critical reliance of the methodology on prompt data reporting. Delays in reporting from these countries led to delayed signals issued after peak periods, highlighting the imperative for enhanced data transmission mechanisms and a more robust review process to mitigate such occurrences.

Additionally, systematic delays observed in countries like Cameroon underscored the importance of integrating contextual information with epidemiological data for a comprehensive assessment of the epidemic situation. Contextual factors such as health worker strikes, exemplified by those in Equatorial Guinea, could significantly influence the interpretation of data and the classification of signals. As highlighted in the outbreak analytics literature, the integration of contextual information with quantitative signals enhances situational awareness and supports more nuanced public health decision-making (28). In countries like Cameroon, contextual nuances such as regional conflicts or resource constraints were critical in maintaining or issuing alerts despite apparent epidemiological improvements. These factors emphasize the necessity for a flexible system that considers real-time operational contexts alongside raw epidemiological data.

Importantly, every week, the WHO AFRO IMST was informed of where data reporting was delayed, allowing the identification of countries needing support despite an absence of an epidemic concern classification. This proactive approach was crucial, as the global situation classification system developed by WHO headquarters often down-classified countries without consulting the regional office, overlooking nuanced local realities.

### Impact of the omicron variant and system adaptations

As anticipated, the predictive performance of both Resurgence and Alert signals declined during the period (T2) heavily impacted by the Omicron variant. However, subsequent adjustments to the SOC classification criteria managed to restore and, in some cases, enhance its compromised performance. These adjustments underscore the significance of operational agility in fast-evolving health emergency response systems, necessitating adaptive strategies to uphold effectiveness amidst dynamic challenges.

One of the major adaptations was the addition of the “Alert (Reduced) Concern” classification. This category allowed limited resources to be redirected without giving a false signal of “low or no risk.” It also clarified the need to continue some reduced non-pharmaceutical interventions (NPIs) and vaccination campaigns.

However, one major drawback of the SOC indicators was their inability to capture nuanced variations in health systems capacity and the actual impact of epidemiological situations across countries, particularly during prolonged waves with high peaks such as those due to Omicron variants. While the criteria demonstrated reasonable effectiveness in categorizing countries based on their needs to receive support, the indicators had an inherent bias toward prioritizing countries with stronger health systems and better coordination in response activities that supported timely data reporting to the regional office. Conversely, in countries with relatively small populations, certain criteria proved insufficient in accurately reflecting changes in the epidemiological landscape over time.

A notable example is Seychelles, an island nation with an estimated population of less than 100,000 people. Throughout the study period, Seychelles consistently remained in the “Very High Incidence” category for most weeks, as its incidence rate exceeded 500 cases per 100,000 people. However, this classification was predominantly influenced by Seychelles’ proactive approach to detecting COVID-19 cases and its small population size, which posed challenges to achieving lower incidence rates. This misclassification created political tensions due to the negative economic and social impacts of what was actually a highly effective response to the epidemic. A similar, though less pronounced, issue was observed in Mauritius, but not in Sao Tome and Principe or Comoros, all of which are relatively small island countries. Differences in COVID-19 testing and case management capacity, sociodemographic characteristics influencing disease dynamics, vaccination rates, and the implementation of NPIs contributed to these variations (29).

A key limitation of this analysis is the lack of a retrospective sensitivity assessment examining the impact of changes in SOC classification criteria over time, particularly the fixed threshold of >500 cases per 100,000 population. Given that the epidemiological context and data availability evolved during the pandemic, it is possible that earlier or alternative thresholds may have yielded different performance outcomes. A formal comparison of historical data against alternative thresholds and indicator combinations could provide useful insights into the robustness and adaptability of the SOC approach. This type of retrospective modeling would help to inform optimal threshold setting in future applications of the system.

Another limitation of this analysis is the variability in country-level data completeness and timeliness, which likely influenced the performance of the SOC system. Countries with irregular reporting patterns—due to technical challenges, personnel turnover, or shifting priorities—were more prone to mismatches between alerts and on-the-ground epidemic trends. These mismatches typically manifested as delays in alert issuance or missed signals. In contrast, countries with consistent and timely data reporting yielded more reliable alert classification. While a formal exclusion-based sensitivity analysis was not feasible due to the dynamic and continuously updated nature of the database, we recognize that performance metrics are partially contingent on data quality. Similar challenges in timeliness and data completeness have been documented in other settings (30), highlighting the critical role of reliable reporting and the need to invest in robust data systems to ensure the effectiveness of real-time surveillance tools, particularly in resource-constrained settings.

### Implications and recommendations for future surveillance

In summary, implementing SOC indicators during the COVID-19 pandemic has yielded several key insights. Firstly, to effectively support resource allocation within constraints, operational systems must comprehensively capture the myriad factors contributing to countries’ successful pandemic responses. This necessitates a holistic approach that goes beyond basic epidemiological data to include factors such as testing rates, health systems capacity, and data reporting capacity. Secondly, as the pandemic transitioned from emergency to routine surveillance, a sustainable, long-term strategy for assessing ongoing epidemic situations became critical. While the SOC indicators were effective in identifying countries requiring emergency support during the global crisis, adapting the methodology and criteria for unique local contexts is essential, particularly with the shift toward sub-national surveillance. This shift underscores the need for real-time epidemiological tools that are adaptable to evolving surveillance contexts, particularly at subnational levels (31). Continuous and timely updating of classification criteria to reflect changing epidemiological realities and pressures on surveillance and case management capacities is paramount.

Moreover, the SOC tool has demonstrated the pivotal importance of enhancing data quality, flow, and surveillance capacity across countries. For any regional assessment to adequately prioritize countries in need, collective motivation among countries to enhance surveillance practices is indispensable. Lastly, evaluation studies assessing the impact of such tools on public health outcomes, including morbidity and mortality, are crucial for advocating their continued utility. By demonstrating the tangible benefits of SOC classifications, policymakers and stakeholders can make informed decisions regarding their integration into ongoing pandemic response efforts and future public health strategies.

## Conclusion

This retrospective analysis of the implementation of the SOC indicator-based operational decision-making system during the COVID-19 pandemic demonstrates its valuable role in supporting timely decision-making and resource allocation across the WHO African Region during the pandemic. Despite inherent challenges such as data delays and evolving epidemiological dynamics, it showed high specificity and reasonable operational utility, particularly after adapting to the Omicron variant context. By addressing its limitations and incorporating contextual adaptations, the system holds promise for enhancing epidemic intelligence and support data-driven responses in both national and sub-national settings, especially in resource-limited environments.
